# Fatherlessness, sperm donors and ‘so what?’ parentage: arguing against the immorality of donor conception through ‘world literature’

**DOI:** 10.1136/medhum-2021-012328

**Published:** 2022-04-25

**Authors:** Grace Halden

**Affiliations:** Department of English, Theatre and Creative Writing, Birkbeck College, University of London, London, UK

**Keywords:** cultural history, literary studies, English literature, Medical humanities, medical ethics/bioethics

## Abstract

Is biology and knowing biological ancestral information essential to the construction of identity? Bioethicist James David Velleman believes this is the case and argues that donor gamete conception is immoral because a portion of genetic heritage will be unknown. Velleman is critical of sperm donation and the absence of a biological father in donor-assisted families. His bioethical work, specifically the 2005 article ‘Family History’, is oft-cited in articles debating the ethics surrounding gamete donations and diverse family formations. However, I wonder to what extent Velleman’s ethical stance is exhibited in contemporary culture? Velleman suggests that innate knowledge of bio-superiority helps readers and audiences appreciate the importance of biological family structures in literature and film; he says, ‘When people deny the importance of biological ties, I wonder how they can read world literature with any comprehension’ (2005, 369). Velleman understands the stories of Oedipus, Moses, Telemachus and Luke Skywalker as demonstrating a universal cultural comprehension that genetics are essential to identity construction. I adopt Velleman’s list of stories and ask if they really can support an antidonation sentiment and suggest that most of the stories actually support diverse family structures. By exploring the significance of story-telling in cultural understandings of family and identity, it is possible to identify the ways in which story-telling can impact how society negotiates complex issues such as assisted reproduction, donor conception and donor industry regulation.

‘Luke, you're going to find that many of the truths we cling to depend greatly on our own point of view.’

(Lucas, 1983)

Bioethicist James David Velleman asks: ‘When people deny the importance of biological ties, I wonder how they can read world literature with any comprehension’ (2005, 369). This thought was expressed in his 2005 article ‘Family History’, in which he argues that both genetic parents should raise their offspring. For Velleman, biological ties and ancestral roots are essential for identity construction; consequently, he argues that donor conception is immoral because a portion of genetic heritage will be unknown, resulting in a ‘disadvantaged’ individual.1 Velleman describes the ideal child-rearing environment as including both genetic and cultural inheritance and thus he privileges heteronormative family models over other family formations.

The publication of ‘Family History’ fits in with a range of work from other philosophers who also argued for the nuclear family as an ideal and relied on ancient stories to support their position (see Browning 2003 and 2007, Almond 2006; Austin 2007). The surge of publications idealising the nuclear family coincided with writings about the popularisation of assisted reproductive technologies (ART) for diverse family construction. Velleman’s arguments against donor conception found companionship in this space. Although many academics provide counterarguments to Velleman’s (see Di Nucci 2016, Roache 2016, Golombok *et al*. 2017; Roth 2016), his work continues to carry weight in articles on ART and cannot be overlooked nor dismissed without challenge. While I disagree that donor conception is immoral, rather than philosophically debate the morality of donor conception, I want to focus on the historical and literary stories Velleman cites in his work and make a different intervention in bioethics. Specifically, I want to think about how we use and produce origin narratives in a world which includes donor-conceived people. Story-telling is important to Velleman who introduces ‘Family History’ by detailing stories of his ancestry and arguing that donor-conceived children have truncated life narratives. However, as we shall see, since 2005 groups like the Donor Conception Network (DCN) have encouraged transparency over donor conception and subsequently a new type of story-telling has emerged that celebrates new family formations and diverse origin stories.

By considering the stories of Sophocles’ Oedipus (circa 429 BCE), Moses (from the Book of Exodus, circa 1400 BCE), Telemachus (attributed to Homer’s *The Odyssey*, 700 BCE) and Luke Skywalker (*Star Wars*, 1977–2019)—examples he calls ‘world literature’—Velleman presents us with the opportunity to explore the significance of story-telling in cultural understandings of family and identity. While Velleman’s antidonor conception stance is unconditional, his reference to literature allows us to further consider how society negotiates complex issues in ART, such as, for example, industry regulation. However, while Velleman uses these narrative examples to argue for the ethical importance of biological ties, I suggest that these texts show that diverse family formations have always existed and that nuclear family models are not necessarily enshrined as idealistic.

I will use Velleman’s examples to challenge three arguments he poses: that identity is best shaped by biology, that donor conception results in disadvantaged children and that donor-assisted families fall short of an ‘adequacy’ standard. I will mainly focus on sperm donation and the importance Velleman places on the biological father in donor-assisted families and within mythical story-telling. To do this, I will concentrate on Velleman’s *Star Wars* example as the saga—often described as a contemporary myth—stretches from 1977 to 2019 and straddles important developments within the donor conception industry including the birth of the first in vitro fertilisation (IVF) child in 1978, concerns over cloning in the 1990s and donor industry regulatory shifts in the 2000s.2

## Story-telling

‘A long time ago in a galaxy far, far away’ (Lucas 1977).

For Velleman, offspring have an ‘inborn nature’ that reflects the ‘natures’ of their biological parents. Consequently, a heterosexual couple rearing their biological progeny will be ‘naturally prepared’ for child-rearing. Furthermore, biological parents will be able to care for their child ‘with sympathetic understanding’, and they will be able to show the child ‘how to recognise and reconcile some of the qualities within itself’ (Velleman 2005, 371). The significance of genetic attachment, for Velleman, extends to the construction of a wider narrative of self through appreciation of ancestral heritage: ‘My family history provides an even broader context, in which large stretches of my life can take on meaning, as the trajectory of my entire education and career takes on meaning in relation to the story of my ancestors’ (Velleman 2005, 375–6). According to Velleman, offspring raised without both biological parents will have narratives to draw on, but will experience feelings of incompletion (Velleman 2005, 376).

In supporting the idealism of the nuclear family model, Velleman remarks: ‘First comes love, then comes marriage, and then the proverbial baby carriage. Well, it’s not such a ridiculous way of doing things, is it?’ (2005, 370). These nostalgic words from a playground nursery rhyme colloquially called ‘The Kissing Song’ are not ridiculous, but this song does present a universality of heteronormativity to the exclusion of LGBTQ+ and solo-parent families. Velleman also universalises the ‘audience’. It is notable that Velleman does not analyse these stories, but rather focuses on how the audience presumably receives and interprets the themes of family and identity. Velleman asks, if not for the importance of biological heritage:

How do they (the audience) make any sense of Telemachus, who goes in search of a father he cannot remember? What do they think is the dramatic engine of the Oedipus story? When the adoptive grandson of Pharaoh says, “I have been a stranger in a strange land,” do they take him to be speaking merely as an Egyptian in the land of Midian? (Velleman 2005, 369)

However, the stories of Oedipus, Moses and Telemachus are open to interpretation and while Velleman presents these works as quintessential examples of the importance of fathering, they can also be read as articulating the significance of non-biological connections to identity construction.

On the surface, Sophocles’ Theban plays depict a man, Oedipus, searching for his biological family and the tragedy of unknown lineage resulting in unwitting incest and patricide. This reading is the one Velleman wants us to accept. However, this tale does not represent positive biological bonds and the importance of being raised by both biological parents. The patricide/incest tragedy would have been avoided if his biological parents had not attempted infanticide. The name Oedipus means ‘swollen foot’ and represents how his parents intended him to be murdered: tied to a tree by his wounded ankles and exposed to the elements (Stewart 2020, 140). The tragedy would also have been avoided if Oedipus had not become consumed with searching for biological truth. The story is paradoxical: Oedipus’ compulsion to discover his genetic lineage leads to tragic consequences but in knowing the truth he may have avoided disaster. Oedipus is a problematic example that will send any analysis in circles.

Moses is perhaps a better example. As Velleman notes, in Exodus 2.22 Moses says, ‘I have been a stranger in a strange land’ and, indeed, his heritage as a Hebrew among Egyptians defines his story. But Moses is also adopted. His adoption by the Pharaoh’s daughter sets Moses on a path that leads him to rescue the Israelites. Moses does not reunite with his birth family and his birth mother is not mentioned again. When the Lord speaks to Moses he says, ‘Who gave man a mouth, or who makes (one) dumb or deaf or seeing or blind? Is it not I, the Lord? […] I will be with your mouth, and I will instruct you what you shall speak’ (Exodus 4:11–12). The Lord here is creator and nurturer and plays a more central role than an earthly parent. In Exodus, complex issues surround the political and ancestral links to Israel; but without adoption, Moses would not have become Judaism’s pre-eminent prophet.

The story of Telemachus is more fitting for Velleman because it details a son earnestly waiting for his father to return from a long quest. However, all three ancient texts deal with illegitimacy, adoption and biological parentage in diverse ways as these mythical characters are from opposing philosophical traditions; to suggest that biology provides centrality to identity is a low-resolution way of understanding the historical and sociopolitical contexts of these stories. Mythical form contributes to an idea of what family can mean, and we can use legends and fiction to explore family formations and identity while still appreciating that they do not promise to reveal a universal ‘truth’. In Velleman’s ‘Narrative Explanation’, an article on story-telling, he argues that an audience ‘understands the narrated events, first, because it *knows how they feel*, in the sense that it experiences them as leading it through a natural emotional sequence’ (2003, 19). However, the audiences for these stories span different times and cultures and can therefore form different inferences as to what the stories convey.

Classical Greece had different terms to differentiate between offspring who are conceived lawfully in marriage (*gnêsioi*), conceived illegitimately (*nothos*) and adopted (*poiêtos*), with priority given to healthy blood offspring (Wilgaux 2011, 212–230). Therefore, heredity is important for our ancient characters. However, it is problematic to assume that the importance of lineage in these stories equates to an exclusive concern with biological family construction. For while Classical Greek sources present the importance of blood lineage, monogamy, marriage and heterosexuality, the constitution of the family was more complicated. Described as ‘a yarn of many strands’, families in ancient societies often included members beyond the nuclear formation (Morgan 2011, 2233). The term ‘household’ was often used instead of the term ‘family’ as a more encompassing word to describe how people were grouped as kin and this included adoptees, slaves, concubines, tenants and visitors alongside the biological family unit, with these structures varying according to class and location (city/rural) (Rawson 2011, 5). Conflating texts from different genres, periods, geographical origins and religions cannot convey a global truth about identity construction, biological kinship and fathering.

In their study of ancient family formations of the Mediterranean, Hubner and Ratzan (2009) note that the trend of fathering offspring later in life meant that many fathers died when their offspring were young, and this loss precipitated the loss of status, property, wealth and rights (Hubner and Ratzan 2009, 10, 22). As Golden (2009) notes, father absence in ancient literature and ancient Greek society could have varied ramifications for offspring; for example, father absence due to the rejection of an illegitimate child is very different from having a deceased father (Hubner and Ratzan (2009, 44)). The upheaval of sudden father absence is seen in *The Odyssey*, in which the loss of the father causes contextually unique issues (including an influx of men attempting to seize the throne, interventions by the Gods and the plot to murder Telemachus). All these problems are more disruptive than Telemachus’ so-called identity issues.

In fact, in Velleman’s examples, offspring would have been at a greater disadvantage—some dead—had they not found alternative families. In Sophocles’ *Oedipus Rex* (the first of his three Theban plays), Oedipus survives his abandonment because he is adopted. In Exodus, the Pharaoh’s daughter sees beyond biological roots to raise a Hebrew child as her own and, in doing so, paves the way for him to become a prophet who leads the people of Israel to freedom. By choosing these examples, Velleman conveniently overlooks the numerous stories that document inadequate parenting by biological fathers. Greek mythology (from which Velleman retrieves the story of Telemachus and Oedipus) has many tales of unacceptable child-rearing by biological parents. One example is Agamemnon who executes his daughter to appease Artemis and quench his army’s bloodlust (Euripides’ *Iphigenia in Aulis*, 405 BCE). The Torah (from which Velleman cites the story of Moses) also contains examples of questionable child-rearing; one is the story of Abraham whose faith in God is more important than his responsibility as a father. While Moses feels like ‘a stranger in a strange land’, I wonder how Isaac, the son of Abraham, feels in Genesis 22 when his father—on the orders of the Lord—ties him to a sacrificial altar and prepares to slaughter him with his dagger.

## Heritage

‘Friendship shows us who we really are’ (Filoni 2012).

*Star Wars*, an epic space-opera franchise created by George Lucas, is Velleman’s most intriguing case study. I will spend more time unpacking this example because it speaks more directly to the question of donor conception and assisted reproduction. *Star Wars* looks back to the past and mythical formation but also considers its own time and changing sociological notions of how families are formed. Let us look at Velleman’s reference to *Star Wars* in full:

When people deny the importance of biological ties, I wonder how they can read world literature with any comprehension. […] How can they even understand the colloquy between Darth Vader and Luke Skywalker? Surely, the revelation “I am your father” should strike them as a bit of dramatic stupidity — a remark to be answered “So what?” (2005, 369).3

The revelation is not ‘dramatic stupidity’; regardless, I do think that ‘So what?’ is an appropriate response to its context within the saga. If we trace the plot—which unfolds over the franchise’s trilogy of trilogies—we see a rejection of blood and the celebration of friendship.

Luke is the biological son of Jedi Knight Anakin Skywalker and Senator Padme Amidala, but he is raised without biological parents after his mother dies and Anakin falls to the dark-side. Anakin is reborn as alter-ego Darth Vader who is the antithesis of Luke, championing an organisation driven by hatred and seeking the conquest of the universe. Throughout the saga, numerous characters distinguish between Anakin (birth father) and the Sith Lord, Vader.4 So, when Vader says to Luke, ‘I am your father’ in *The Empire Strikes Back* (Kershner 1980) the reaction—in context—must ultimately be ‘So what?’ Luke and his twin sister Leia continue to fight against the Empire after they discover Vader is their father: they ultimately say, ‘So what?’.

Even though—as film critic Andrew Lewis Conn notes—the revelation of Luke’s parentage was shocking to the 1980s audience (a moment which ‘caused a collective frisson’), we—like Luke—need to say, ‘So what?’ and acknowledge the complexity of Luke beyond his genetic line Conn (1997, 2). Indeed, Conn recalls how his 8-year-old self ‘flinched and went cold’ at the line ‘I am your father’ but reconsidered the line contextually—as we all must do—and came to realise that it prepares us for ambiguity not just in the films but in life (Conn 1997, 7). Vader does not just tell Luke about his parentage; the film tells the audience that identity is complicated, and we are asked to what extent this information informs Luke’s selfhood. Possible answers to this question come in the following seven films (plus one animated film, two anthology films, and the television specials and series) as *Star Wars* champions the bond forged between Luke and a family that is chosen rather than born. The non-biological relationships between Luke, Han Solo, Obi-Wan Kenobi, Yoda, R2-D2, C-3PO and Chewbacca are the most significant counters to the threat of the dark-side and provide Luke with the support and strength he needs to fight the Empire.

There is no doubt that the bonds of friendship are all important in the saga (see Littman 2016, 127–135), but I am not suggesting that biology is insignificant. After all, Luke refers to Vader as ‘father’ and fights for the rehabilitation of Anakin. However, although Velleman presents the caveat that ‘biological origins needn’t be worth embracing in order to be worth knowing’ (Velleman (2005, 377)), the quest for identity in *Star Wars* is not the story of Luke discovering his roots, but of Luke assisting Anakin’s rebirth. Jedi Master Yoda describes the biological connection between Luke and Vader in *Star Wars: Episode VI Return of the Jedi* (1983) as a burden because the revelation of parentage has more meaning to Anakin’s journey than Luke’s. There is significance in Charles Taliaferro and Annika Beck’s claim that Vader never actually takes on a fatherly role with Luke despite their genetic link: ‘Luke seems to be acting more like the father than Vader, as Luke tries to turn him back to “the good side,” as if the villain is a lost child.’ As Taliaferro and Beck note, Anakin/Vader as a father operates as a ‘toxic caricature of love’ and **‘**a painful portrait of how fatherhood can go wrong’ (Taliaferro and Beck (2016, 118)). Contrary to the ideal Velleman articulates, in which both genetic parents must raise a child, in *Star Wars* the galaxy breathes a sigh of relief that Vader did not raise his twins.

So far, I have focused on Velleman’s example: one isolated moment in the second film in the franchise. Now, I want to explore how identity is addressed in the later films because, although they do not figure for Velleman, in the later examples we can see changing perspectives on the family which coincide historically with developments in the field of assisted reproduction. This symbiotic relationship helps us think about how we understand family stories related to donor conception.5 The third *Star Wars* trilogy (*Star Wars: Episode VII The Force Awakens* (Abrams 2015), *Star Wars: Episode VIII The Last Jedi* (2017), and *Star Wars: Episode IX The Rise of Skywalker* (2019)), sees the introduction of Ben Solo (Kylo Ren), who is the son of Luke’s sister Leia and Luke’s friend Han Solo. Ben is born with the Skywalker predisposition to the Force (a metaphysical power), which negatively affects his childhood. The weight of identity expectations and biological lineage damages him and he struggles to live up to his namesake ‘Ben’ Kenobi, his mother the Resistance leader, his hero father, his uncle (Luke) and his grandfather (Anakin, aka Vader). Tormented by the pull to both the light and dark sides of the Force and unable to navigate his complex biological heritage, Ben embraces the identity of Kylo Ren and turns against his family, even murdering his father.

Ben Solo is accused of simultaneously being too biologically informed and not genetically shaped enough. The Resistance views him as ‘too Vader’ and the First Order as ‘too Skywalker’. In *The Last Jedi*, Supreme Leader Snoke states that when he first encountered the boy, he sensed ‘the potential of your bloodline; a new Vader’, then moments later mocks him by saying, ‘you have too much of your father’s heart in you’ (Johnson 2017). It is of little wonder then that at the centre of Ben’s identity crisis is his claim that he has not had space to develop as a unique person:

Choice? I have no choice and never did. Even my name isn’t a choice. The dark side and light both claimed me for their own the moment I was born […] Whether it’s Skywalker or Snoke, neither one sees me as a person. I’m just a… legacy. Just a set of expectations (Soule and Sliney 2020, 9).

The choice to name himself Kylo is significant. His new name combines Skywalker and Solo—two names that he considers burdensome—but pruned to remove their authority over him. The resulting murderous desire to cement himself as both Kylo Ren and Supreme Leader of the First Order reflects his desperation to establish his identity despite his lineage.

Meanwhile, a new character called Rey is rising as a Jedi Master. During the saga, Rey discovers that her grandfather is Sheev Palpatine (aka Darth Sidious), the Sith Emperor. This revelation is jarring but does not sway her to darkness. She rejects what is presented as her ‘blood right’ to become Empress and instead fights for the Resistance. In a significant scene, Kylo Ren and Rey both try to wield Anakin Skywalker’s lightsabre—a weapon to which Ren believes he has a birthright as Anakin’s grandson. Yet, the weapon favours Rey and is wielded by her. The weapon does not favour the biological Skywalker line, and this exchange symbolically highlights the fallacy of presenting ‘blood’ as superior to any other sort of connection.

In the final moments of the last film, a stranger asks Rey for her name and she announces that it is Skywalker. Despite Palpatine’s blood running through her, she adopts the name and heritage that fits her blossoming identity. By the end of the film, the only person who bears the Skywalker name is not a biological descendant. Biology is not all important and consequently, when Palpatine tells Rey ‘long have I waited, for my grandchild to come home!’, the revelation is met with dismissal because Rey is a Skywalker: it is something she is emotionally and psychologically, not something she has in her veins. Luke makes this point himself when he tells her that ‘some things are stronger than blood’ (Abrams 2019).

The Force is a good analogy for how we can perceive identity. The Force is cultural, ancestral, taught, adopted and universal: it is described as ‘an energy field created by all living things’ that exists ubiquitously. The Force is described as energy, but it is also attuned at a cellular level: ‘Midi-chlorians are a microscopic lifeform that resides within all living cells’, and they communicate with the Force *(* Lucas 1999). While Yoda tells Luke in *Return of the Jedi* that ‘the Force runs strong in your family’, this same Force runs strong in Yoda, Kenobi, other Jedi Masters and Sith Lords (Marquand 1983). The Force is also presented as beyond biology, and this is reinforced in *Rogue One* (Edwards 2016) when Chirrut Îmwe becomes one with the Force and engages in masterful combat despite his blindness. When Rey and Luke train together in *The Last Jedi*, Luke encourages Rey to close her eyes, breathe and feel to experience the Force. As Elizabeth F. Cooke remarks, ‘The Jedi way has much in common with a kind of mind-body dualism, whereby one must overcome his biological nature rather than become unified with it’ (Cooke 2005, 92). So, while biology may have an influence, the Force—which represents environmental and spiritual connection—is more significant.

Bearing this in mind, the Force—an environmental influence—is more instrumental to identity construction than genetics. The exchange between Jedi Qui-Gon Jinn and Anakin’s mother Shmi reveals as much. Having identified that the Force is strong in young Anakin, Qui-Gon enquires about the boy’s father. However, Shmi reveals that she is a solo mother and the child is fatherless: ‘There was no father. I carried him. I gave birth. I raised him. I can’t explain what happened’. The famous Skywalker name came not from the paternal, but the maternal line.6 Qui-Gon rationalises that Anakin was miraculously ‘conceived by the midi-chlorians’ (inferring that the male gamete component was donated) (Lucas 1999). He also suggests that Anakin is shaped by the universe and that Anakin’s identity is not located in blood. The characters of Anakin and Luke change through their experiences with both their biological families and their non-biological families. Anne Lancashire is right when she argues that in *Star Wars*, ‘Individual human lives (and political systems) are not predetermined by “destiny” but are defined and redefined by difficult and continually recurring moral choices’ (Lancashire 2000, 37).

## The disadvantaged child

‘A Chosen One shall come, born of no father’ (Gray 2019).

)For Velleman, donor conception is immoral because it produces a disadvantaged child who does not know their biological roots. Velleman equates donor-conceived babies with babies who experience gestational complications, and he suggests that donor-conceived people are ‘handicapped’ (Velleman 2005, 372). Velleman additionally argues that a parental bond may be difficult to forge without this biological connection: ‘I would not want to have raised my younger son without having known my maternal grandfather, with whom he has so much in common. I would never have understood my older son if I hadn’t known his uncles, on both sides’ (Velleman 2005, 370).

However, in *Star Wars*, many characters are unaware of their genetic history for a multitude of reasons: taken as children to be trained by the Jedi (Qui-Gon and Obi-Wan), abandoned by irresponsible parents (Han), orphaned in the galactic war (Jyn Erso), cloned (Boba Fett), misled about their genetic history (Leia, Luke, and Rey) or produced through fatherless donor conception (Anakin). *Star Wars* is populated by heroes who have little or no access to their full ancestral history. This detail reflects those in society who do not have full awareness of their bloodline for various reasons, including abandonment, estrangement, adoption, parental death, family breakdown, domestic violence, relocation, donor conception, displacement, migration and incomplete ancestral history. Coming from a family of academics and teachers has enabled Velleman to imagine that he follows in his ancestors’ footsteps. By leaning heavily on his family history when considering his identity, Velleman glorifies detailed awareness of lineage to which many do not have access. Further, Velleman’s assertion that identity is shaped by ancestral knowledge prioritises nuclear family models as comprising of ‘full’ identity, while other models engender suboptimal partial ones.

In the chapter ‘Persons in Prospect’ from *Beyond Price*, Velleman speaks of a ‘standard of adequacy’ to justify procreation (Velleman 2015, 107). The ‘inadequacy’ of donor conception is ‘that a life estranged from its ancestry is already truncated’ (Velleman 2015, 108). However, it seems that Velleman is not speaking of *adequacy* but instead of the idealism of an ‘advantaged’ child. It appears that the recommended *ideal* is for a child to be born to ‘healthy’ biological parents who can offer a ‘stable’ environment with access to complete ancestral information. New family models, such as donor-assisted families with solo parents or same-sex parents, are therefore described by default as inadequate. Velleman challenges the morality behind ‘new ideology’ that allows the construction of new family models: ‘According to the new ideology of the family, of course, virtually any adult is in a position to satisfy this requirement, since a family is whatever we choose to call by that name. But this new ideology is precisely what I am questioning’ (Velleman 2005, 374). Velleman questions what family means and, in turn, interrogates the impact new family forms have on the successful construction of identity.

To address this question, I must examine not just Velleman’s fictional examples but the difference between father-absence and fatherlessness, as well as the donor conception industry itself. I will first unpick the difference between absence and fatherlessness—two things Velleman conflates. In the stories Velleman uses, offspring deal with father-absence—this means that each character should have a biological father in their life who socially, culturally and legally is expected to take responsibility for their offspring after choosing to conceive within a lawful marriage. Oedipus is born to married parents who abandon him; Moses’ parents hide their son and then send him down the Nile in the hope of saving his life; Telemachus’ father leaves the home for war and endures a difficult return journey; and Anakin Skywalker, having attempted to murder his wife, has his children taken from him. In all four examples, offspring are conceived within a heterosexual marriage in which the biological father might be expected to help raise the child. The absence of the father causes problems purely because this expectation is not met.

Psychologists Silverstein and Auerbach (1999) make two points about modern neoconservative ideas of the family that connect with ancient thought on the role of the family patriarch. They note that the suggestion that fatherless families are likely to be impoverished and struggling is ‘buttressed by unconscious gender ideology and traditional cultural values’. They also note that research reveals ‘that neither mothers nor fathers are unique or essential; and that the significant variables in predicting father involvement are economic, rather than marital’. Velleman’s examples do not reveal the importance of knowing a genetic parent, but they instead testify to the importance of how the patriarch connects the family to inheritance, status, wealth, class and community. Silverstein and Auerbach (1999) also explain that the difficulties faced by children from father-absent families—such as divorced families—are due to the significant disruption of the child’s environment (such as visitation, change of living conditions, etc) rather than the absence of one biological parent (Silverstein and Auerbach 1999, 399).

The child conceived via sperm donation does not face the same pressures found with father-absence (such as divorce and death), which involve the sudden loss of a biologically linked patriarchal figure expected to assume legal and social responsibility. The sperm provider is not expected, nor obliged, to occupy the role of the father; instead, fathering is assumed by a male raising the child, or non-existent in the case of lesbian couples or solo mothers who form fatherless families. The complexity of fatherlessness in sperm donation narratives is not compatible with the father-absence narratives Velleman offers in his articles.

Velleman and I also read the donor conception industry very differently. Partly, this is symptomatic of the times about which we are writing. Velleman (despite more recent pieces) is writing about sperm donation within the American model of donor conception pre-2005, which was plagued by unregulated practice, donors with complete anonymity and children raised with no knowledge of their conception history. In *Offspring,* Barry Stevens (2001) highlights the plight of donor-conceived children born under these conditions. Stevens, a donor-conceived person, discovered later in life that his biological father was an anonymous gamete donor; while searching for his donor, he discovered the unregulated practices at the London Barton Clinic in the mid-twentieth century. During his investigation, Stevens confronted the possibility that his donor could have sired hundreds of offspring. This scenario is presumably what Velleman is imagining; however, the industry has changed significantly since the birth of Barry Stevens in 1952.

Although industry regulation will not alter Velleman’s antidonor position (indeed his stance was unchanged in *Beyond Price*, 2013), it is important to note how industry regulation continues to differ across the globe, and in the USA, from state to state. While Velleman refers only to the US model, I also invoke the UK model which, in terms of regulation, has modelled tighter practices since 1991. Since Velleman’s first article on donor conception in 2005, guidance in the USA and the UK has become clearer. For example, the US Food and Drug Administration (FDA), Human Fertilization and Embryo Authority (HFEA), DCN, Donor Sibling Registry (DSR), and American Society for Reproductive Medicine (ASRM), etc advise against the scenarios Velleman articulates as characteristic of the industry. The UK and US industries recommend that prospective parents select a registered donor from a regulated bank with donor-identity release. In contrast to Velleman’s depiction of the industry, the ASRM advises that parents should raise donor-conceived people from infancy with knowledge of their donor conception, parents should select a traceable donor, and the parents should obtain as much data as possible on the donor (The Patient Education Website of the American Society for Reproductive Medicine).

Guidance for best practice in gamete conception has been articulated for a while; for example, the DCN was formed in 1993 to support donor-conceived families and encourage openness about donor conception (see Montuschi 2013). In UK law, all registered donors are limited to assisting 10 UK families; they must provide detailed medical and personal information to help the construction of a profile, and since 2005 they must agree to their identity being shared with the offspring when they turn 18. The limitations imposed in the UK mean that it is improbable that a registered donor will create hundreds of offspring—in fact, UK statistics suggest that ‘less than 1% of donors create 10 families with most sperm donors creating one or two’ (Human Fertilisation and Embryology Authority).

In America, regulated sperm donors are also subject to robust screening; America’s largest sperm bank (California Cryobank) accepts only 1% of donor applicants, and competitor Xytex accepts less than 3%. Following the ‘donor eligibility final rule’ enforced in 2005 by the US’s FDA, donor samples are screened for ‘clinical evidence of relevant communicable disease agents and diseases’ (FDA 2018). Since then, biological data are more readily available; many banks test donors for over 280 genetic and inherited conditions (example: Xytex, since 2019). A consensus has emerged that argues against complete anonymity in donor conception; many academics, industry professionals and donor-conceived offspring argue for known donation or identity release (see Allan 2017).

Shifts in regulation, the importance of seeking known donors (or ID release donors), and the recommendation for complete transparency with offspring from an early age have culminated in increased attention being given to how donor conception is narrativised and the importance of story-telling to include new family formations. The UK’s DCN states: ‘The DCN takes a clear stance that supports openness in families and thus the majority of members are parents who have undertaken to tell their children, often from a very early stage in their development’ (Pettle and Burns n.d., 15). By prioritising the act of story-telling in families created through technologised reproduction and donor-assisted conception, the DCN hopes to not only diversify how conception is taught to young people but to normalise this diversity. Their *Telling and Talking* series includes guides, booklets and pamphlets aimed at sharing the donor story with friends and families, and their *Our Story* series is aimed at children and depicts different types of conception journeys (including sperm donation, egg donation, double donation, embryo donation and surrogacy). While Velleman suggests donor-conceived offspring are lacking narrative structures, there is increasing awareness that narrativising the donor conception journey is crucial to helping children understand themselves and articulate their unique contexts. These children do not lack a narrative; they have a different narrative and the significance of this is addressed within these emergent modes of story-telling.

Considering the seismic shift in how leading organisations understand best practice in the donor conception industry, and the recommendations for openness, story-telling and transparency, it is critical to distinguish between donor-conceived people who are born following the guidelines set out by the HFEA (*et al*) and those who are raised without knowledge of their conception history, without access to data and without the option of donor release (Stevens, eg). When in 2011 bioethicist Margaret Somerville drew on Velleman’s ‘Family History’ to agree that donor conception is harmful and therefore against the Hippocratic oath, she was only considering examples of bad practice. In her paper she refers to two antidonation case studies by adult donor-offspring, extrapolating from those the ‘fact’ that ‘most don’t even know they’re donor-conceived’ (Somerville 2011, 280).7 Like Somerville, I can provide two stories from donor-conceived people to support my argument; both people were raised with the knowledge of their donor conception. First is Carly, daughter to a solo mother, who explains, ‘I’m proud of being donor conceived as I feel wanted because I know my Mum had to go to more effort than most parents to have me’ (DCN 2020). Second is Kate, born to a lesbian couple, who states:

My parents started talking to me about being donor conceived when I was about four […] Genetics has never been very important to me – I know there’s a craze at the moment for getting your genes ‘tested’ but it’s not something that has ever appealed. I tend not to be convinced by the notion that DNA is what comprises a family (DCN 2018a).

Despite concerns articulated by Velleman and Sommerville, more contemporary studies have shown that new donor-assisted families are healthy ones. As Parke (2013) notes, despite ‘dire predictions’, the reality is that: ‘The children and their parents who form these new families are thriving and flourishing just as well as naturally conceived families’ (Parke 2013, 140). One study found that donor-conceived teenagers are no different to those born into ‘natural conception families’ and concludes that ‘children born through reproductive donation are, by necessity, planned and there is evidence to show that planned pregnancies are associated with more positive psychological outcomes for mothers and children’ (Golombok *et al.* 2017, 1973–4; see also Roth 2016, 42).

While Velleman does not engage with regulatory change, the regulations introduced in the UK have encouraged a general shift in cultural attitudes towards gamete conception and related ideas of what constitutes a parent and family. Respected groups like the Nuffield Council on Bioethics (NCB) have attempted to redefine contemporary definitions of terms like ‘kin’ and ‘reproduction’ for use beyond the heteronormative nuclear family. For example, the NCB suggests that the nuances of donor conception require a new way of defining terms like donor and father, which are conflated in Velleman’s work: ‘The “recipient” parent or parents will be the child’s real parents from the beginning […] the law makes provision for the donor to be excluded from the legal status of parent’ (Montgomery *et al*. 2013, xvii). Academics are also redefining key terms when discussing new parenting models; in *Diverse Pathways to Parenthood* (2020), Damien Riggs argues that the term ‘reproduction’ needs to be defined beyond genetic connections:

I understand the term “reproduction” broadly, not limited to genetic reproduction. Specifically in regards to becoming parents, humans do not simply reproduce themselves. At a genetic level, those who conceive a child are producing a human life, and in a sense are reproducing the pattern of life and death that shapes the human world, but they are not reproducing themselves (Human Fertilisation and Embryology Authority).

Riggs’ (2020) assertion here disagrees with Velleman’s claim that ‘if I want to know what a person *like this* can make of himself, I can look first at what my parents and grandparents made of themselves’ (Velleman 2005, 368).

The narrative examples Velleman claims illustrate that ‘people unacquainted with their origins’ have been viewed ‘as dramatically, even tragically disadvantaged’ are outdated and not entirely contextually accurate (Velleman 2005, 369). Despite Velleman republishing ‘Family History’ in *Beyond Price* in 2013 and writing a book on identity in 2020, he has not responded to the evolution of the donor conception industry; consequently, his concerns respond to an outdated idea of donor conception. Not only has *Star Wars* moved on—shifting from Luke to Rey—but the industry has progressed from secrecy to transparency.

## Regulation

‘In the Force, very different each one of you are’ (Bullock 2008).

*Star Wars* draws on a rich and ancient culture of mythical story-telling. It is not surprising to see similarities between *Star Wars* and the mythical examples Velleman refers to, including the themes of the displaced child, adoption, and unknown and rediscovered biological parentage. However, while the *Star Wars* saga is set in a ‘galaxy a long time ago’, it is a product of the 1970s and the development of the franchise coincides with the ART revolution.

The first film of the saga was released in the 1970s; a decade marked by crisis and hope. Miles Booy traces how the predicaments of the 1970s (Vietnam, Watergate, the illegal surveillance of citizens by the CIA, the energy crisis, etc) informed the making of *Star Wars* and argues that the films respond to ‘the changing cultural landscape’ (2021, 22). I agree with Booy that the 1970s was a ‘polarising’ time, and this polarity is also reflected in how the ART industry was being perceived following the birth of Louise Brown—the first IVF baby in 1978. Susan Golombok notes that this decade was a pivotal time in Western culture for how families were perceived: ‘Changes to the structure of the family have been taking place since the 1970s’, and these changes coincided with changing attitudes towards people identifying as LGBTQ+ and the growing gay rights and women’s liberation movements (Golombok 2015, 2–3). The birth of Brown was followed by IVF success in America in 1981 (2 years before *Return of the Jedi*). The growth of the industry, as evidenced by the increase in fertility clinics in the 1980s and 1990s, coincided with a torrent of breakthroughs in ART (such as greater success with frozen gametes and embryo transfers) and cultural shifts that welcomed treatment for same-sex couples, co-parents, solo people and unmarried couples.

By the end of the 1990s, Vader was dead, Anakin Skywalker was born fatherless to a solo mother, and many countries had prohibited completely anonymous gamete donations. The emergence of debate around the problem of anonymity coincided with the development of the *Star Wars* franchise, which centred on the secretive birth of the Skywalker twins who were raised without knowledge of their parentage. The twins’ birth featured in the 2005 film, *Revenge of the Sith,* the same year that the UK introduced compulsory identity release for all gamete donors and banned completely anonymous donations. ART development after 1970 spanned the three *Star Wars* trilogies and ran in parallel to the saga’s complex treatments of solo parenting, anonymous parentage, donor conception, bioengineering and the welcoming of diverse family formations.

When Vader announces ‘I am your father’ in the 1980 film he signifies the older tradition of glorifying genetic parenthood and blood lineage (that is still with us); however, Luke in the 2019 film signifies changing times by passing the family mantle to Rey, to whom he has no genetic ties. From Shmi’s solo motherhood to fatherless Anakin, to the creation of a clone army, the franchise has responded to the bioethical debate which coincided with its development. If, as Silvio and Vinci (2007) argue, the influence and endurance of *Star Wars* positions the franchise as not just a work that draws on myth and can be described as mythical, but a work that is itself a cultural artefact, then we should consider how the franchise coincides with ART development as an ‘ideological investment’ (Silvio and Vinci 2007, 3).

How *Star Wars* responds to the ART industry is complex, perhaps fittingly given that Lucas used ART to have his own child via surrogacy. The control the Empire has over clones may, on the surface, suggest the demonisation of reproductive technology. *Star Wars* does not present a ‘black and white’ thesis on reproduction but instead ambivalently explores how technology can be both positively and problematically used. *Star Wars* presents a middle ground that Velleman’s article overlooks, but one that I and many advocates of donor conception welcome: the need for industry regulation to prevent the abuse of ART without imposing such extreme regulation that diverse family formations are oppressed. To explore this ‘middle ground’, I will look at how reproductive technologies are presented in the saga.

In *Attack of the Clones* (Lucas 2002), Obi Wan Kenobi discovers a clone army created from the genetic template of Jango Fett who donated his genetics in exchange for a son. This contractual agreement mirrors common practice in many clinics in which ART costs are reduced if gametes are shared. In this film, the clone army is cinematically depicted as sectioned into square military units and equipped with body amour featuring a dome-shaped helmet; this presentation resembles rows of test tubes containing gametes, divided into square or rectangular trays ready for freezing. The presentation of multiple identical offspring in *Attack of the Clones*, and the birth of the Skywalker twins in *Star Wars: Episode III Revenge of the Sith* (2005) articulate anxieties about the increased probability of multiples (twins, triplets, etc) following stimulated intrauterine insemination and multiple embryo transfer in IVF. In fact, the link between multiple pregnancies and ART led to the introduction of the One at a Time campaign by the HFEA in 2007 to limit multiple pregnancies following a spike in the 1990s and 2000s. So, on the surface, the clone trope may suggest criticism and concern over ART.

However, clones do not represent an argument against reproductive technologies and donor conception wholesale but instead represent the urgent need for industry regulation. If anything, Jango’s donor storyline warns against unregulated practices and reflects the ‘Wild West’ nature of American ART in the twentieth century, in which one unregistered donor could anonymously sire hundreds of children. Simply put, Jango would have been able to create a baby army in 2002 in America but not in 2005 in the UK. *Star Wars* coincided not only with the ART revolution but also with its regulation and the debates surrounding the ethics of donor conception.8

The Empire’s creation of clones, and the monstrous visages of their principal characters (Darth Vader, the Emperor, General Grievous, Darth Maul, Supreme Leader Snoke) have obvious parallels to societal concern over the use of ART technologies to create hybrids, chimeras and genetically engineered super-people. Concern over irresponsible genetic engineering was a heated topic in the mass media in the 1990s following the ground-breaking news that The Roslin Institute had successfully cloned a sheep, Dolly. A year later, in 1997, Stuart Newman, a member of the Council for Responsible Genetics, alongside biotechnology critic Jeremy Rifkin, attempted to patent the speculative creation of an animal/human hybrid known as Humouse. Their ambition was not to put the patent to use themselves; instead they wanted to prevent others from creating such ‘monsters’ by reserving the technology as intellectual property. We can easily see context that informed the 2002 film *Attack of the Clones*. It is also easy to see how we could misread the saga as advocating for exhaustive regulation. However, while regulation is important (no one wants a million Jango Fetts), the extreme of the Empire’s rule is what happens when regulation is not in the interests of people and does not allow for the formation of diverse family structures.

The Empire represents the potential recklessness of reproductive technologies through the construction of a clone army, yet the Empire’s tyrannical control over the population of the galaxy marks the danger of excessive regulation. The Empire presents two extremes at once: irresponsible reproductive science and discriminatory regulation. Many scenes in the first *Star Wars* trilogy were shot in Tunisia—notably, scenes for the Skywalker home planet of Tatooine. In Tunisia, fertility treatment for solo mothers and same-sex couples is illegal. In the 1970s, when the first film was released, Tunisia did not have a licensing body and had only a few clinics in comparison to the exponential growth of the industry in America. However, even now in 2022 Tunisia, ART is restricted to married couples due to religious and cultural beliefs. On Tatooine (described as outside the reach of the Republic), the tyrannical rule of the Empire is felt, and under this control communities are marginalised, families are broken apart and populations are wiped out. The Empire represents a new type of life under the power of the Sith: one of struggle, restriction and the complete loss of freedom. This is what discriminatory regulation looks like.

Under the Republic, there is hope. It was within the Republic that Anakin was born to a solo mother and was prophesied to be the Chosen One; and it was within the Resistance (which emerges after the collapse of the Republic) that Rey was able to choose a family rather than be confined to her genetic one. *Star Wars* presents the audience with a ‘middle-ground’ in which diverse families are welcomed but non-discriminatory regulation of reproductive technology is recommended. This is similar to the ‘middle-ground’ the FDA, HFEA, DCN, DSR and ASRM advocate. The extreme Velleman presents is incompatible with the pro-diversity message *Star Wars* enshrines. As Obi-Wan warns Anakin, who is falling into the darkness due to his inability to compromise and empathise with the views of others: ‘Only a Sith deals in absolutes’ (Lucas 2005).

## New myth

‘Your focus determines your reality’ (Lucas 1999).

‘Always remember’, Qui-Gon advises a young Anakin Skywalker, ‘your focus determines your reality’ (Lucas 1999). A prophetic line: Anakin will one day focus on the dark-side of the Force and, despite his efforts to protect his family, become the architect of their destruction; in essence, his focus on darkness becomes his reality. Qui-Gon’s words also have resonance when we think about how narratives are interpreted, what themes we focus on, and what messages we infer. In ‘Narrative Explanation’, an article on event narration and story-telling, Velleman speaks of ‘projective error’ in which, regardless of plot comprehension, an audience can find sense on ‘emotional terms’. Here he cites the story of Oedipus again and argues that while the plot may be considered ‘a mere absurdity to those of us who don’t believe in fate’, we can understand emotionally because ‘we know the feeling of being undone by our own efforts’ (Velleman 2003, 21). In ‘Family History’ we have numerous examples of what I would consider ‘projective error’, in which Velleman interprets legendary texts through his belief in the importance of biology in the establishment of identity. From this he concludes donor conception must surely be immoral.

In this article, I have used the same texts to suggest another position. We both could be said to be guilty of indulging what Velleman describes in ‘Narrative Explanation’ as ‘the confusion between emotional and intellectual instances of “Aha!”’ when reading mythical stories (Velleman 2003, 22). In his work on narrative understanding, Barwell (2009) draws on Velleman’s narrative work, and argues that epics, novels, plays and films may contain narratives but also that ‘typically, readers and watchers must supply some of the explanatory connections’. Therefore, he argues, ‘It is to be expected that different readers and spectators will use the same text to produce different emplotments’ (Barwell 2009, 58). Yet, I do not wish to conclude by suggesting that Velleman and I simply read these stories differently and thus must ‘agree to disagree’. Instead, I want to turn again to the power of ‘new mythology’ and how contemporary texts, like *Star Wars*, should propel us towards a deeper appreciation of how our cultural conceptions of family are expanding.

Missing from Velleman’s article are the voices of donor-assisted families, for which story-telling is an important tool for transparency and awareness. In accordance with recommendations by groups including the HFEA and DCN, donor-assisted parents are encouraged to explain to children from infancy about their donor-conceived background. The DCN publication *Choosing to be open about donor conception: the experiences of parents* advises early and regular ‘telling’ (Pettle and Jan, 8). Books including *The Pea That Was Me* by Kimberly Kluger-Bell (2012) and *Our Story* by the DCN (2018) encourage the normalisation of donor conception to children. The dominant cultural narrative of the nuclear family ideal is shifting, and this shift is nicely articulated in Hoffman and Asquith’s, *The Great Big Book of Families*, which opens with an idealised picture of a nuclear family consisting of a father, a mother and two children; surrounding the picture are the words: ‘Once upon a time most families in books looked like this. But in real life, families come in all sorts of shapes and sizes’ (Hoffman and Asquith 2010, 1). The DCN supplies resource packs for schools to help ‘encourage children to think about and share with pride, facts about the special people in their life’. By talking about their own family constructions, the class can then ‘talk about how there are all kinds of different families and that children can be raised by people who aren’t genetically connected to them’ (DCN 2018b, 4). Donor conception features in many young adult books (see: Sarles 2021) and in numerous memoirs by solo mothers by choice (see Mattes 1997; Morrissette 2008; Roberts 2019).

The question should not be whether donor conception is moral (I believe it is, Velleman disagrees … the debate will endure), but a question about the importance of story-telling to our understandings of family and identity. Velleman’s reliance on Oedipus, Moses, Telemachus and Skywalker—and indeed his other philosophical work on narratives—suggests that story-telling is vital to our interpretation of selfhood. Therefore, it is incoherent to overlook the significance of donor conception story-telling and how these stories also articulate contemporary conceptions of what constitutes a family. Looking at ART in contemporary story-telling through memoirs, blogs, podcasts and interviews must inform how bioethicists approach the donor conception debate. For as the NCB argue: ‘Donor conception is first and foremost about people […] any debate about the ethical considerations that should inform public policy on donor conception should start, not with the analysis of abstract principles, but with the people concerned, and the reality of their lives’ (Montgomery *et al.* 2013, xix).

There are many counterarguments to the idea that an ‘advantaged’ child is one raised by both biological parents. Some of these counterarguments come from mythical story-telling, testimony from donor-conceived persons and statistical data. I am concerned that by questioning the morality of ‘the new ideology of families’, new family types (often Queer) will be increasingly marginalised while the heteronormative nuclear family model is further privileged. I am also concerned about the misrepresentation of cultural artefacts like literature, myth and film as historically idealising the nuclear family as this is not a transparent way of presenting the complexity of family models that have existed since antiquity. Velleman’s suggestion that donor-conceived offspring have a fractured narrative does not appreciate the diversification of narratives for different families. The question should not be about the significance of biology to identity construction but about the importance of how we talk about different types of origin stories. Unwillingness to enfold diverse conception narratives into the story of reproduction is what will disadvantage children, no matter how they are conceived, born and raised.

## Data Availability

All data relevant to the study are included in the article or uploaded as supplemental information.

## Notes

1. Donor conception includes sperm donation, egg donation, double gamete donation and embryo donation. Donation can involve natural insemination, artificial insemination, intrauterine insemination and in vitro fertilisation. Here, I focus on sperm donation as David J Velleman’s bioethical work mainly focuses on the importance of the biological father.

2. It was not appropriate or possible to involve patients or the public in the design, or conduct, or reporting or dissemination plans of my research.

3. Luke and his sister are raised without knowing their biological parents but only the importance of the biological father receives sustained attention. Ahuva Cohen addresses this gender problem in *Star Wars* when she says, ‘“I am your mother, Luke,” said no *Star Wars* character ever’ (Cohen 2016). Beyond the Luke/Vader dynamic, other father/son relationships are highlighted in the saga including Ben/Han Solo and Jango/Boba Fett. Further, when the Jedi remove Anakin Skywalker from his mother’s care to educate him in the Force, they imply that the influence of male Jedi is more instrumental to identity construction than contact with a biological mother. Charles Taliaferro and Annika Beck attribute Anakin’s obsessive tendencies with being an only child to a solo mother and therefore (inadvertently perhaps) idealise the father within a heteronormative family construction to the healthy development of identity: ‘he [Anakin] doesn’t have to share his mother’s love, and so he doesn’t learn how to feel anger or grief in the course of emulating a good father in a healthy family’ (2016, 118). The saga assumes that boys are like their fathers and girls like their mothers (as McDowell (2016) notes when comparing Leia to her mother (2016, 99)). Since the 1970s, the saga has produced non-progressive examples of what mothering and fathering entail with prioritisation given to the influence of the father. Similarly, although Velleman highlights the importance of both biological parents to the construction of a whole identity, he overtly dislikes solo mothers by choice, saying ‘the serviceability of single parenting cannot justify the creation of children with the intention that they grow up without fathers of any kind’ (2005, 75). When Velleman compares conceiving a donor-conceived child with ‘taking a teratogenic medication’ during pregnancy, he suggests some women have ‘an obligation not to conceive’ (2005, 73–4). The importance of the *Star Wars* line, ‘I am your father’, in Velleman’s work and the relationship between his other literary examples like Telemachus/Odysseus reductively prioritises paternal influence on offspring identity construction.

4. The Emperor makes a distinction between Anakin and Vader in *The Empire Strikes Back* (1980) as does Kenobi in *Return of the Jedi*. In *Revenge of the Sith* Yoda describes Anakin as ‘consumed by Darth Vader’ and in *Return of the Jedi*, Vader claims that the name Anakin ‘no longer has any meaning for me’.

5. Velleman’s first article was published before the sequel trilogies; the latter films further evidence that identity construction is more complex than Velleman assumes.

6. Of note here is the significance of gestational ties to identity construction. It is ambiguous whether Shmi is the biological mother of Anakin or if she is a gestational surrogate. Egg-donation, embryo-donation and gestational ties (especially in surrogacy debate) are overlooked in Velleman’s work, his literary examples, and often in antidonor debate.

7. Margaret Somerville’s article represents work from that period that reflects the experiences of donor-conceived people raised without knowledge of their donor conception background and who were entering adulthood in the early 2000s.

8. The saga’s cloning storyline undermines Velleman’s argument that biology is the seat of identity. The television series *Star Wars: The Clone Wars* (2008–2020) references differences in personality, behaviour, and psychology across the clones as highlighted by Yoda when he says, ‘In the Force, very different each one of you are’ (Bullock 2008).
